# Perceived stigma in AI-generated mental health imagery: an online study

**DOI:** 10.1192/j.eurpsy.2025.1505

**Published:** 2025-08-26

**Authors:** J. Grimmer, N. Khorikian-Ghazari, L. Schoch, N. L. Hartmann, A. Hasan, I. Papazova

**Affiliations:** 1 Department of Psychiatry, Psychotherapy, and Psychosomatics, University of Augsburg, Medical Faculty; 2 DZPG (German Center for Mental Health), partner site München/Augsburg, Augsburg, Germany

## Abstract

**Introduction:**

Artificial intelligence (AI) has enriched the everyday lives of many people and is used in a variety of ways, i.e. as a resource of medical information. One of the most popular AI functions is the generation of images of real-world settings. Recent research (Mei et al. ACM FAcct 2023; 1699-1710) has shown, that AI could reinforce prejudice and can be biased in its answers leading to stigmatization in the online world as well. This has dramatic consequences as stigma is known to affect mental health negatively (Pérez-Garín et al. Psychiatry Res. 2015; 228 325-331). Little is known, however, if and to what extend AI generated images reveal bias towards people with mental illness or corresponding institutions such as psychiatric clinics.

**Objectives:**

The aim of this exploratory study is to investigate whether AI-generated images of psychiatric institutions, scenes, and severe mental illnesses are perceived as stigmatizing compared to other hospital scenes and severe illnesses from patients, mental health experts, and the general population in Germany.

**Methods:**

Two researchers prompted three different AIs to generate various realistic medical scenes (prompts: person suffering from a severe mental illness, person suffering from a severe illness, mental health institution, hospital, psychiatric ward, hospital ward, incident in a mental health institution, incident in a hospital, electroconvulsive therapy session, cardiopulmonary resuscitation session). For each chatbot, one image per prompt was selected randomly for the following online survey. In a mixed subject design, participants were randomly assigned to one of three groups displaying the generated images of one AI. Then, they were asked to rate the images on SAM rating scales, adjective scales, to provide a title for the image, and to decide whether the image stigmatizes specific groups. The survey starts on November 2024 and a total sample size of 100 subjects is aimed for.

**Results:**

Preliminary results will be presented at the congress.

**Conclusions:**

This study examines the effects of AI-generated images on patients, experts and the general population. It attempts to find out whether and to what extent AI-generated images stigmatize people with severe mental illnesses and to what extent psychiatric institutions are portrayed realistically compared to general medical institutions and severe illnesses.

**Disclosure of Interest:**

J. Grimmer: None Declared, N. Khorikian-Ghazari: None Declared, L. Schoch: None Declared, N. Hartmann: None Declared, A. Hasan Consultant of: Rovi, Recordati, Otsuka, Lundbeck, AbbVie, Teva and Janssen-Cilag, Speakers bureau of: Janssen-Cilag, Otsuka, Recordati, Rovi, Boerhinger-Ingelheim and Lundbeck, I. Papazova: None Declared

